# Generic Learning-Based Ensemble Framework for Small Sample Size Face Recognition in Multi-Camera Networks

**DOI:** 10.3390/s141223509

**Published:** 2014-12-08

**Authors:** Cuicui Zhang, Xuefeng Liang, Takashi Matsuyama

**Affiliations:** Graduate School of Informatices, Kyoto University, Kyoto 606-8501, Japan; E-Mails: zhang@vision.kuee.kyoto-u.ac.jp (C.Z.); tm@i.kyoto-u.ac.jp (T.M.)

**Keywords:** multi-camera network, person re-identification, small sample size, generic learning-based ensemble, optimal solution, diversity/accuracy dilemma

## Abstract

Multi-camera networks have gained great interest in video-based surveillance systems for security monitoring, access control, *etc.* Person re-identification is an essential and challenging task in multi-camera networks, which aims to determine if a given individual has already appeared over the camera network. Individual recognition often uses faces as a trial and requires a large number of samples during the training phrase. This is difficult to fulfill due to the limitation of the camera hardware system and the unconstrained image capturing conditions. Conventional face recognition algorithms often encounter the “small sample size” (SSS) problem arising from the small number of training samples compared to the high dimensionality of the sample space. To overcome this problem, interest in the combination of multiple base classifiers has sparked research efforts in ensemble methods. However, existing ensemble methods still open two questions: (1) how to define diverse base classifiers from the small data; (2) how to avoid the diversity/accuracy dilemma occurring during ensemble. To address these problems, this paper proposes a novel generic learning-based ensemble framework, which augments the small data by generating new samples based on a generic distribution and introduces a tailored 0–1 knapsack algorithm to alleviate the diversity/accuracy dilemma. More diverse base classifiers can be generated from the expanded face space, and more appropriate base classifiers are selected for ensemble. Extensive experimental results on four benchmarks demonstrate the higher ability of our system to cope with the SSS problem compared to the state-of-the-art system.

## 1. Introduction

Recently, surveillance video cameras have been widely established in both public and private places with the purpose of security monitoring, access control, *etc.* Video surveillance systems typically monitor an environment by a multi-camera network, which comprises a set of vision sensors [1]. Each camera has its own processing element and memory. Different cameras can communicate through a network. Person re-identification is a necessary, but challenging task in multi-camera networks, which aims to determine if a given individual has already appeared over the camera network, and to re-identify this person at different locations and time instants. Although variant biometric traits, such as gait, can be used to recognize subjects, it is preferred to use more distinct traits, such as faces. Face recognition has become a hot research topic in multi-camera network-based applications.

The development of robust and accurate face recognition algorithms in multi-camera networks is not an easy task, due to several issues, including: (1) hardware system limitation. Considering the cost and computational complexity, each camera has limited processing elements, while multiple cameras are often non-overlapping (disjoint). The same subjects observed in multiple camera views may undergo significant visual appearance changes. Additionally, there are (2) un-constrained image capturing conditions. In real-world scenes, un-cooperative subjects can be recorded by different cameras in arbitrary poses, expressions, in different illuminations, viewpoints and with other environmental changes.

Appearance-based recognition has been acknowledged as one of the most popular approaches for face recognition, such as eigenfaces, Fisherfaces, Bayes matching and their weighted, kernelized and tensorized variants [2–4]. However, these methods require a large number of training samples during the training period. Given sufficient training samples per subject, they can have a very high recognition accuracy (e.g., 90%). However, in multi-camera networks, it is very difficult to collect adequate training samples due to the limitation of the hardware system and the complicated image capturing conditions. The available training samples for each camera are usually very limited. The small number of training samples compared to the high dimensionality of the face space leads to a serious problem, the “small sample size” (SSS) problem, which challenges existing recognition algorithms severely. A considerable amount of efforts have been devoted to solving the SSS problem [5–11]. This problem is interesting to both theoreticians and practitioners, because the SSS problem can easily contaminate the design and evaluation of a proposed recognition system. An extreme case of SSS is single sample per person (SSPP) [12], where only one sample is available for each subject.

### 1.1. Challenges of SSS Face Recognition

**Figure 1.**
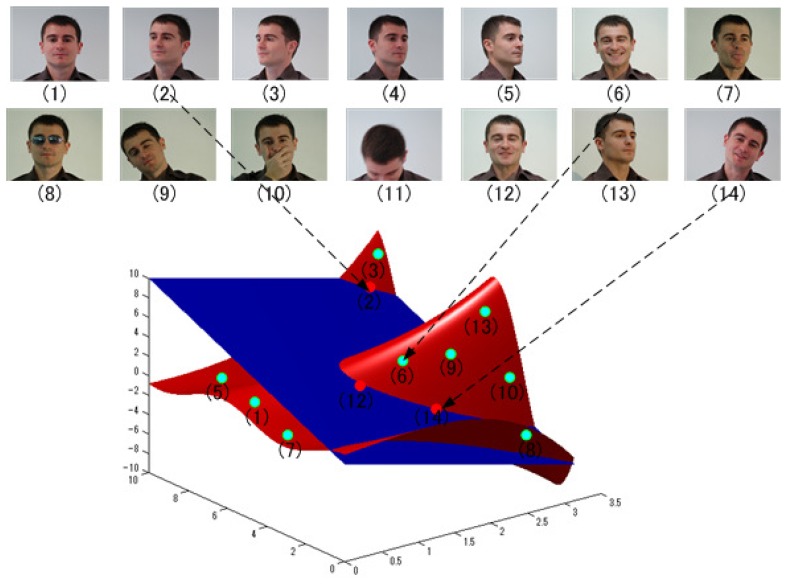
The demonstration of two manifold surfaces: The red one represents a full face space projected from all of the samples of one person; and the blue one is a face space learned when only three samples are available.

The SSS problem is illustrated in Figure 1, where the red manifold surface reveals a face space projected by plenty of samples of a person, and the blue one represents the learned face space from a small number of samples of him. With insufficient samples, the estimated face space is very likely to overfit the small training data, and the real face space cannot be well represented. Conventional methods suffer serious performance degradation from the small training data compared to large appearance variations caused by occlusions, aging, illumination changes, head poses, facial expressions, *etc.* [13–15].

To address the SSS problem, there have been many attempts in the literature such as the random subspace [5], patch-based methods [16], *etc.* However, the trained classifiers from small data are likely to become unstable and have poor generalization ability [9]. Currently, a machine-learning technique, known as ensemble learning, has received considerable attention and has led to significant improvement in recognition accuracy. Such algorithms are based on the idea that a pool of different classifiers can offer complementary information for classification. They first generate multiple base classifiers and then aggregate them based on an ensemble rule. They benefit from the base classifier collaboration, where the overall discriminative power is higher than a single classifier.

In the context of ensemble, accuracy and diversity are two important parameters referring to the performance of an ensemble system. Diversity measures the disagreement degree in the output of a set of base classifiers [17]. Intuitively, there is not a clear accuracy gain in an ensemble built from a set of identical base classifiers. The ideal situation is that a set of base classifiers can bear with different errors, and the final classification error can be minimized by the combination of multiple base classifiers. However, existing ensemble methods often suffer from an underlying problem that the species of base classifiers learned from insufficient training data are not diverse enough to form an adequate feature space. They are very likely to be tightly correlated and make similar errors. Having less diverse base classifiers has become a bottleneck of existing ensemble algorithms.

**Figure 2.**
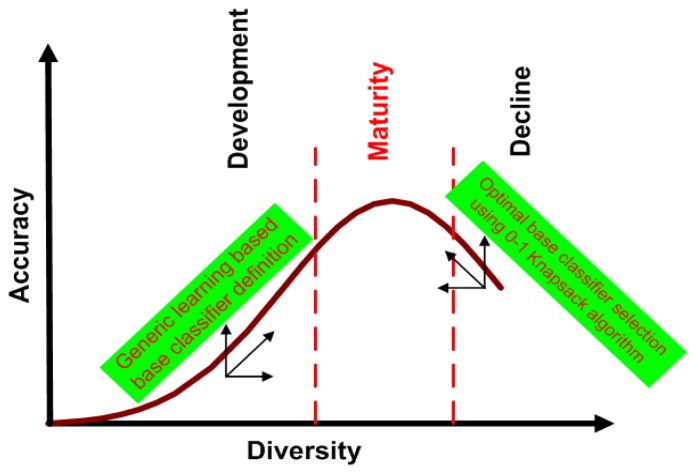
The relationship between diversity and accuracy, which is summarized in three stages: development, maturity and decline. This figure is plotted based on the experimental results shown in Figure 9.

Even having a large number of diverse base classifiers, another problem is worth further discussion. We cannot guarantee that all generated base classifiers are appropriate for classification. Integrating inappropriate base classifiers may degrade the performance. The relationship between accuracy and diversity is illustrated in Figure 2, where the growth of accuracy and diversity can be classified into three stages: development, maturity and decline. The accuracy grows up with the increase of diversity in the development stage, halts at the maturity stage and then decreases, while the diversity keeps increasing in the decline stage. We can see that the increase of diversity can improve the accuracy to a certainty degree, but they are not always linearly related. This leads to the diversity/accuracy dilemma [17], which is a challenging problem for existing ensemble learning algorithms. To solve the diversity/accuracy dilemma, it is highly desirable to improve both the diversity and the accuracy in the development stage and to suppress the unexpected diversity in the decline stage. Inspired by this finding, this work proposes a novel generic learning-based ensemble framework (GL-E), which arguments the small training data by generating new samples to generate diverse base classifiers during the development stage and introduces a tailored 0–1 knapsack solution [18] to select appropriate base classifiers for ensemble in the decline stage, so that the final accuracy and diversity are located at the maturity stage.

### 1.2. Contributions

Compared to existing algorithms [5–11], GL-E has the following characteristics:

- GL-E augments the small data by yielding new samples. From the enlarged training data, more diverse base classifiers can be generated than existing ensemble methods;
- Conventional ensemble algorithms involve all base classifiers for aggregation, which leads to a diversity/accuracy dilemma. In contrast, GL-E selects just appropriate base classifiers for ensemble based on an optimal solution. This strategy achieves the trade-off between the diversity and accuracy.

The rest of this paper is organized as follows: Section 2 reviews some related works. The motivation of this work is stated in Section 3. Section 4 describes the proposed method in detail. We evaluate the system using four widely-used databases in Section 5. Finally, Section 6 concludes this paper.

## 2. Related Works

While this paper mainly concerns the SSS problem, we provided a literature review on ensemble learning-based methods for SSS face recognition. They can be mainly divided into three categories: (1) global feature selection using random subspace; (2) patch (block)-based local feature extraction; and (3) global and local feature integration.

The first category introduces a random subspace to ensemble. Literature work [19] finds that strong and stable base classifiers defined by subspace-based algorithms (e.g., Linear Discriminant Analysis (LDA)) are not suitable for ensemble rules. Thus, a random subspace (RS) [5] was employed to generate weak, but diverse base classifiers by doing random sampling in the PCA subspace. As RS focuses on the global-rather than local-based feature extraction, local discriminant information is not guaranteed. Motivated by this, the second category develops local feature extraction-based methods by partitioning face images into multiple patches/blocks and extracts features from each patch/block separately. These are named the patch (block)-based methods [9]. An early attempt [20] divided face images into six elliptical sub-regions, such as nose, mouth and eyes [12], and learned a local probabilistic model for recognition. Topcu *et al*. [16] proposed an alternative way of partitioning face regions into equally-sized small patches. Features extracted from each patch were classified separately, and the recognition results were combined by a weighted sum rule. As small patches usually have limited ability to deal with large variations, considering that global and local feature representation can provide complementary information, the third category integrates the global and local features for classification. For instance, Su *et al*. [21] developed a hierarchical model, where the global feature vector is extracted from a whole face image using the low frequency Fourier coefficients, and the local feature vectors are formed based on LDA. The global feature and local features are combined by a weighted sum rule. Zhu *et al*. [9] proposed a multi-scale patch-based collaborative representation (MPCRC) by integrating the complementary information obtained at different scales of patches. Spreeuwers *et al*. [8] proposed a 30-region method, which defined 30 regions of variant sizes according to experimental experience. The largest one covers almost the whole face region.

Beyond SSS face recognition, there have been some special attempts at SSPP face recognition. For instance, unsupervised learning-based methods apply the classical PCA and its variant extensions, such as Two-Dimensional PCA (2DPCA) [22], Projection-Combined Princi-pal Component Analysis ((PC)^2^A) [23] and 2-Directional 2-Dimensional PCA ((2D)^2^PCA) [24], to generate a holistic-feature vector from the total gallery data. Virtual sample generation-based methods, e.g., SVD-LDA [25], generate some virtual samples for each person. Generic learning-based methods, e.g., adaptive generic learning (AGL) [26] and adaptive discriminant analysis (ADA) [10], collect an auxiliary generic set containing multiple samples per person to learn the recognition model to identify the people with a single sample. Patch (block)-based methods, such as block PCA [27], block LDA [28] and discriminative multi-manifold analysis (DMMA) [11], are employed to learn base classifiers from local subregions and integrate them to form a final classifier.

## 3. Motivation

Ensemble learning can be performed in either the original training data or the enlarged training data. Generic learning (GL) is an effective approach to augment the small training data. Conventional GL-based approaches utilize an auxiliary set (named the generic set) containing a large number of samples to assist in learning the classification model of small data. Intuitively, the faces of all human beings look alike, which implies that different people may have similar face images. Conventional GL-based methods try to grab face images from the generic set that are similar to those in the sample set and treat them as the samples in the small sample set. However, no matter how similar these sample are, they actually do not belong to the subjects in the small sample set. These methods suffer from a severe problem that if these subjects, who belong to the generic set other than the small sample set, are present in the test sample set, they are very likely to be misidentified as the subjects in the small sample set.

**Figure 3.**
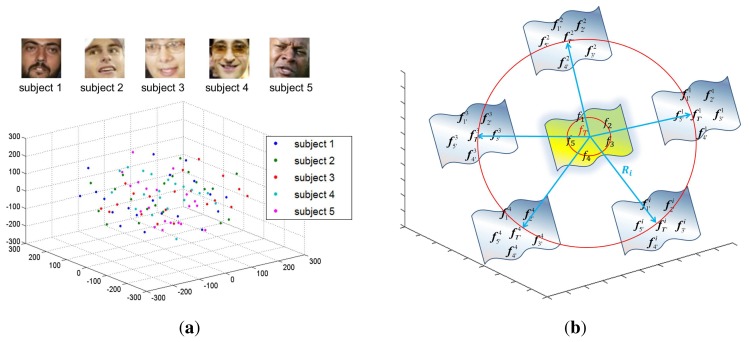
The motivation of this paper: (**a**) Generic distribution of face images in the manifold face space described by the first three coordinates of locality preserving projections (LPP) [29]; Two-dimensional face images are mapped into the manifold face space based on liner embedding; (**b**) Face space expansion by generating new samples based on a generic distribution.

To overcome this problem, this paper proposes augmenting the small training data by generating new samples based on a generic distribution other than grabbing samples from the generic set directly. To explore the generic distribution of face images, we use the YouTube Faces database (YTF), which contains the largest number of subjects (1595 different subjects) and has the highest degree of unconstrained behaviors. Each subject possesses an average of 2.15 videos. Each video ranges from 48 frames to 6070 frames, with an average of 182 frames per video. Faces in each frame were detected by the Viola-Jones face detector. We randomly selected 100 face images owned by five subjects and projected them to the 3D space for the ease of presentation. The distribution of face images is shown in Figure 3a. We can see that all of the face images are uniformly distributed in the manifold face space. Motivated by this observation, for the given small training data, our algorithm aims to generate new samples based on a uniform generic distribution.

The main idea is illustrated in Figure 3b. For the given small training data, which is located at the center of the face space, we introduce several random matrices *R_i_*, *i* = 1, 2, …, *L* based on a uniform distribution to explore the new possibilities of face space. For each random matrix, we can generate a new training sample set. Additionally each training sample set can be used to train a base classifier. Specifically, instead of adding random matrices to the original face images, we first model the feature distribution of the small data by a template image *f_T_* and then add *R_i_* to *f_T_* to get an image $f_{T\prime }^{i}$. Then, quad-tree decomposition [18] is performed on each $f_{T\prime }^{i}$ to get an image encoding pattern, according to which the original face images are re-organized to generate a new training sample set. Since all of the new samples are generated using the original face images, they should be located around the original small sample set. This strategy expands the face space to a large extent.

## 4. The Proposed System

**Figure 4.**
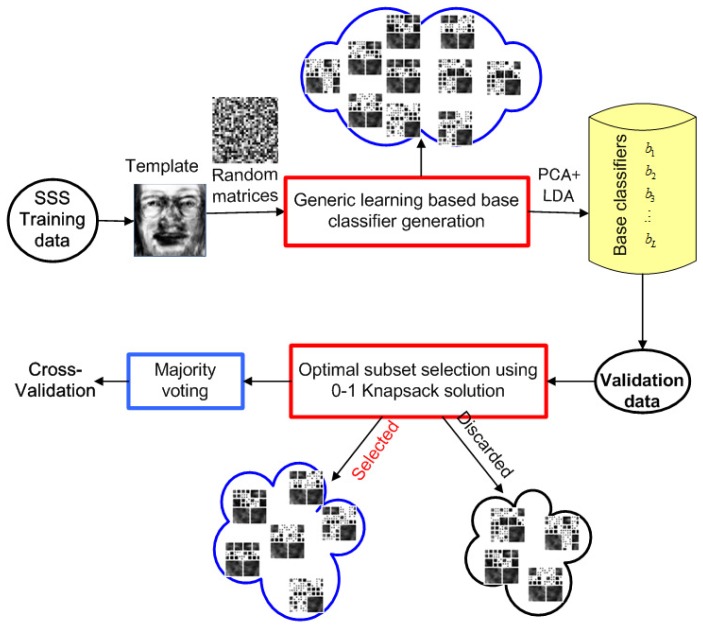
Illustration of the generic learning-based ensemble framework (GL-E).

The whole framework is illustrated in Figure 4, which involves the following three steps:

- Generic learning-based base classifier definition: The face space is expanded by generating new samples based on a uniform distribution. Diverse base classifiers are generated from the expanded face space subsequently.
- Optimal solution-based base classifier selection: To address the diversity/accuracy dilemma, an optimal solution motivated by the 0–1 knapsack problem is employed to select appropriate base classifiers for ensemble.
- Majority voting-based base classifier integration: Multiple base classifiers are integrated based on majority voting.

We design each step in the following Sections 4.1–4.3, respectively.

### 4.1. Generic Learning-Based Base Classifier Definition

The generic learning-based base classifier definition mainly consists of three operations: (1) template image generation and random matrix introduction; (2) new sample generation based on quad-tree decomposition; and (3) the definition of the accuracy and diversity of base classifiers.

#### 4.1.1. Template Image Generation and Random Matrix Introduction

Motivated by the idea of LDA, which encodes discriminant information by maximizing the between-class scatter matrix *S_b_* and minimizing the within-class scatter matrix *S_w_* (see Equation (1)), we define a template face *f_T_* by Equation (2) to represent the discrimination distribution across a database.

(1) $$\{\begin{array}{l} S_{b}=\sum_{i=1}^{c}N_{i}\left(\mu_{i}-\mu \right){\left(\mu_{i}-\mu \right)}^{T}\\ S_{w}=\sum_{i=1}^{c}\sum_{x_{k}\in X_{i}}\left(x_{k}-\mu_{i}\right){\left(x_{k}-\mu_{i}\right)}^{T} \end{array}$$

(2) $$f_{T}=\text{diag}(\frac{S_{b}}{S_{w}})$$

where *c* is the number of classes in the dataset, *μ* is the mean image of all classes, *μ_i_* is the mean image of class *X_i_*, *N_i_* is the number of samples in class *X_i_* and *x_k_* is the *k*-th sample of class *X_i_*. For the face images of size *m* × *n*, the covariance matrix $\frac{S_{b}}{S_{w}}$ is of size (*m* × *n*)^2^. *f_T_* is defined by the diagonal vector of $\frac{S_{b}}{S_{w}}$, whose entries represent the variances at each pixel of the face region across the database. It is of the same size as the original face images.

Since the template image *f_T_* generated from the small training data has a weak ability to represent the whole face space, we generate several new images $f_{T\prime }^{i}=\left\{f_{T\prime }^{1},f_{T\prime }^{2},\ldots ,f_{T\prime }^{i},\ldots f_{T\prime }^{L}\right\}$ by introducing a set of random matrices *R* = {*R*_1_, *R*_2_, …, *R_i_*, …, *R_L_*} to expand the estimated face space. Each random matrix *R_i_*, *i* = 1, 2, …, *L* is distinct and has the same size as *f_T_*, whose elements are randomly chosen from a uniform distribution. The new image $f_{T\prime }^{i}$ is generated by the dot product of *f_T_* and *R_i_* as:

(3) $$f_{T\prime }^{i}=f_{T}\cdot R_{i}$$

**Figure 5.**
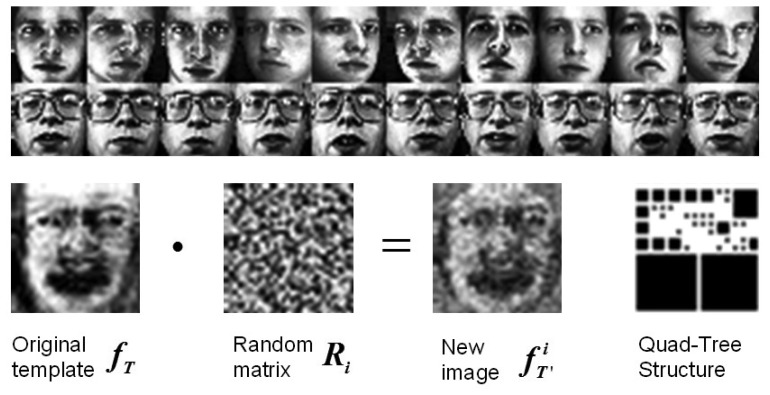
Example of template face generation and random matrix introduction for the Oivetti face database (ORL).

Please note that the generation of *f_T_* fails under SSPP, since no samples are available to construct *S_w_*. Regarding this, *f_T_* is defined by just *S_b_* under SSPP. An example of template face generation and random matrix introduction is shown in Figure 5.

#### 4.1.2. New Sample Generation Based on Quad-Tree Decomposition

We perform quad-tree decomposition on each $f_{T\prime }^{i}$ to study the regions of high density of discriminant features across the face area. The decomposition is performed according to a function *doSplit*(*r*) defined in Equation (4) [18]. If the variance of a region *τ* (starting from the whole region of $f_{T\prime }^{i}$) is higher than or equal to a threshold variance (*t_v_*), then *r* is split into four sub-blocks of the same size. The partition carries on until no blocks satisfying the criterion function or candidate blocks have reached the smallest size.

(4) $$\text{doSplit}(r)=\{\begin{array}{l} \text{true},\text{while}(\text{var}(r)\geq t_{v})\\ \text{false},\text{otherwise} \end{array}$$

**Figure 6.**
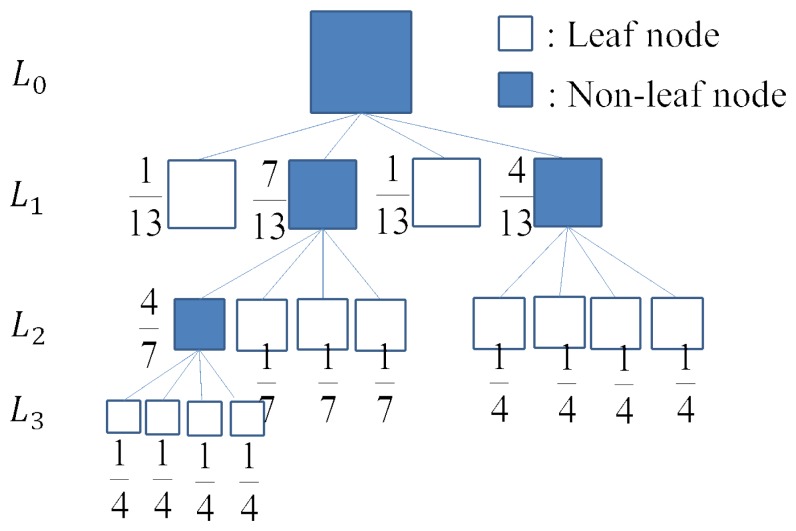
An example of a quad-tree partition.

The image $f_{T\prime }^{i}$ is split into less and bigger blocks for a large *t_v_*, but into more and smaller blocks for a small *t_v_*. Local variances usually vary on different databases. Even for one database, it is rather difficult to find the best partition using one universal threshold *t_v_* [18]. For this reason, this paper develops a data-adaptive threshold definition algorithm utilizing the quad-tree structure. The entire procedure is as follows:

Step 1 Assign an initial estimate for the threshold: *t_v_* = 0.5 * *var*(*wholeR*), where *wholeR* denotes the whole region of $f_{T\prime }^{i}$;
Step 2 Partition $f_{T\prime }^{i}$ using *t_v_* based on Equation (4). This step produces a hierarchical quad-tree structure as shown in Figure 6. It contains leaf and non-leaf nodes. For each leaf node *subR_i_*, we calculate its variance: *var*(*subR_i_*);
Step 3 From bottom to top, we calculate the variance of each non-leaf node *subR_j_* by the average of its four children *subR_k_*, *k* = 1, …, 4 based on a weighted sum rule. The weight of each child node *subR_k_* is defined by the number of leaf nodes in the subtree rooted at *subR_k_* against that number rooted by its father node *subR_j_*. An example of the quad-tree structure with weight assignment is shown in Figure 6. When we reach the top level, we can calculate the value of the root node *var*(*root*) and assign it to the new threshold $t_{v}^{\prime }$ for the quad-tree partition.
Step 4 Repeat Steps 2 through 3 until the difference between two quad-tree partitions is smaller than a threshold distance. Specifically, we encode each quad-tree by a 0–1 sequence according to a full quad-tree partition, where leaf nodes are assigned one and non-leaf nodes are assigned zero. The distance between two quad-trees is defined by the Hamming distance of two 0–1 sequences. If this distance is smaller than a threshold distance denoted by *ε* * *M*, where *ε* is the tolerance error and *M* is the length of the 0–1 sequence, then the iterative process stops. Here, we define *ε* = 5% in this paper.

Our algorithm updates the threshold according to the last quad-tree structure in each repeat. The new threshold is closer to the values of regions containing a high density of discriminant features. Finally, our algorithm can find the most appropriate threshold for each $f_{T\prime }^{i}$ automatically.

**Figure 7.**
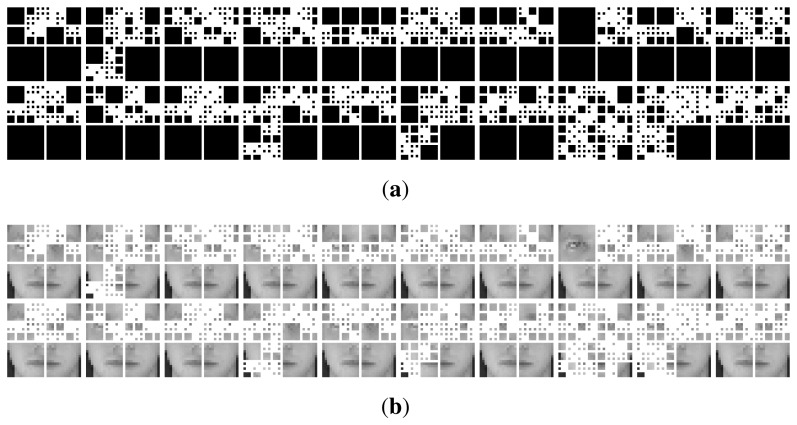
An example of 20 quad-trees: (**a**) quad-trees; (**b**) quad-tree partitions on a face image.

After quad-tree partition, we get a face encoding pattern, as shown in Figure 7a. Each quad-tree structure refers to such a pattern. Given *L* random matrices, we can get *L* such encoding patterns. All face images in the original training set are re-organized according to each pattern to generate a new training sample set. The procedure is as follows: since larger blocks (in black) imply that the density of discriminate information within them is low, these blocks have no need to keep their original sizes. We do down-sampling on them by resizing them to smaller regions of ((*d*/2) × (*d*/*2*)), where *d* is the dimension of the block (in pixels). The smallest blocks (in white) imply that they have a high density of discriminant features. We will keep their size. Quad-tree decomposition and block resizing generate new face samples, which are smaller than the original face images. A base classifier *b_i_* is learned from each new training sample set.

**Figure 8.**
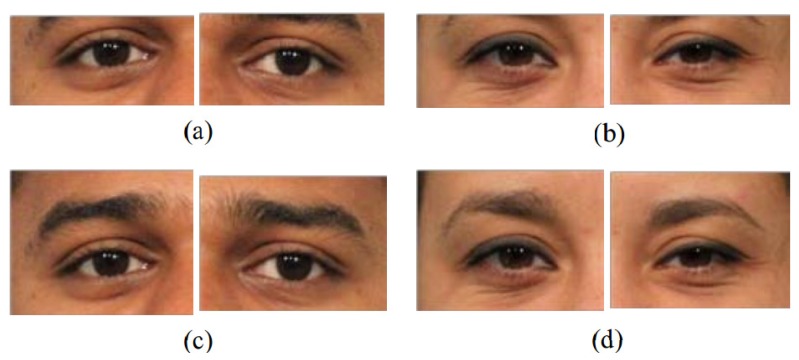
Example periocular images from two different subjects [30]: (**a**, **b**) without eyebrows; and (**c**, **d**) with eyebrows.

A interesting observation is that our quad-tree partitions shown in Figure 7 prove an important literature finding in the biometric field. Recently, researchers have used the area around the eye, the periocular region (see Figure 8), as a stand-alone biometric with promising results [30]. They found that the periocular region offers advantages over full face biometrics, such as: (1) it is least affected by expression variations, aging effects and changes due to the growth of male facial hair; (2) the performance of full face recognition degrades in the presence of pose variations, whereas the periocular region-based identification may perform better in the case of extreme pose changes when only one eye is completely visible; and (3) periocular region-based recognition remains effective even if most of the lower face region is occluded and as long as only one eye remains visible. Currently, these findings have only been observed from experimental results. They have not been proven from other perspectives, yet. Our partition results shown in Figure 7 demonstrate the higher density of the discrimination information in the periocular region as opposed to other regions and prove theses findings accordingly.

#### 4.1.3. The Definition of the Accuracy and Diversity of Base Classifiers

We regard the performance of an ensemble *E* as a function *F*, which corresponds to two parameters: (1) the accuracy (*acc*(*E*)); and (2) the diversity (*div*(*E*)).

Let a labeled training sample set be *S* = {(*x*_1_, *y*_1_), (*x*_2_, *y*_2_), …, (*x_N_*, *y_N_*)}, where *y_i_* is the class label of *x_i_*. The base classifiers *B =* {*b*_1_, *b*_2_, …, *b_L_*} of an ensemble are trained on this set. For each sample *x_i_*, the output of a base classier *b_j_* is *b_j_*(*x_i_*). The accuracy of each base classifier *b_j_* is defined in Equation (5) by the ratio of its correctly-classified samples against the total samples. Additionally, the accuracy of the ensemble *acc*(*E*) is defined by that ratio based on the majority voting of a set of base classifiers in Equation (6).

(5) $$\text{acc}(b_{i})=\frac{\text{num}(\text{correctSamples}(b_{i}))}{\text{num}(\text{totalSamples})}$$

(6) $$\text{acc}(E)=\text{acc}(\text{majorityVote}(b_{i})),i=1,2,\ldots ,L$$

The diversity of ensemble (*div*(*E*)) is investigated in several literature works. For instance, in [17], six common measurements are analyzed, namely disagreement measure, double fault measure, KWvariance, inter-rater agreement, generalized diversity and measure of difficulty. As concluded in [17], these measures are motivated by different problems in pattern classifications. None of them is universal for all applications. In this paper, we utilize the disagreement measurement to calculate *div*(*E*), which was originally proposed in an ensemble framework [31], and the intuition behind its definition coincides with the expectation of our method (two diverse classifiers perform differently on the same training data).

For a query set containing *N* test samples, each base classifier *b_i_*, 1 ≤ *j* ≤ *L* assigns a label to each sample *x_i_*, 1 ≤ *i* ≤ *N*. The diversity between each pair of base classifiers *b_i_* and *b_j_* is defined by the number of samples for which they have different labels against the total number of samples:

(7) $$div_{i,j}=\frac{n(a,b)}{N},a\neq b$$

where *a* and *b* denote the labels assigned by *b_i_* and *b_j_*, respectively; *n*(*a*, *b*), *a ≠ b* represent the number of samples, on which *b_i_* and *b_j_* have different labels. Diversity among all base classifiers is calculated by averaging over all pairwise diversities in Equation (8), which measures the total disagreement among all base classifiers.

(8) $$\text{div}(E)=\frac{2}{L(L-1)}\sum_{i=1}^{L}\sum_{j=1}^{L}{\text{div}}_{ij}$$

### 4.2. Optimal Solution for Base Classifier Selection

**Figure 9.**
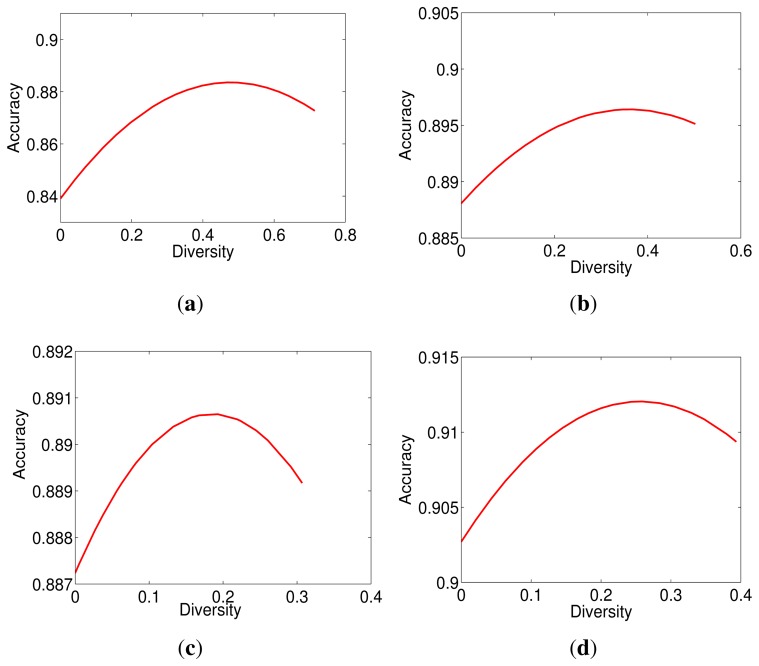
A demonstration of the diversity/accuracy dilemma on four public face databases: (**a**) ORL; (**b**) Yale2; (**c**) AR; and (**d**) FERET.

As mentioned before, not all generated base classifiers are located within the face space, as we expected. Even the base classifiers in the face space may perform differently according to the discriminant features involved. That is, some base classifiers are not appropriate for classification. Integrating inappropriate base classifiers may lead to the diversity/accuracy dilemma, which has been illustrated by the experimental results in Figure 9.

To alleviate this problem, an optimal solution motivated by the 0–1 knapsack algorithm is tailored to select appropriate base classifiers for ensemble. The selection can be considered as a combinatorial optimization task. Suppose each base classifier has two items: (1) the recognition accuracy; and (2) the disagreement with other base classifiers in making decisions; which contribute to the diversity of an ensemble. Given a set of base classifiers, our goal is to find an optimal subset of base classifiers *E* that maximizes the final accuracy, while the diversity of *E* is still higher than or equal to a threshold diversity *t_d_*. The mathematical interpretation of our problem is:

(9) $$\begin{array}{l} \text{max}\left(\text{acc}\left(E\right)\right)\\ \begin{array}{cc} \text{subject to} & D\left(E\right)\geq t_{d} \end{array} \end{array}$$

where *D*(*E*) = *div*(*E*) denotes the diversity of *E* and *t_d_* is the diversity threshold.

Our problem is very similar to the conventional 0–1 knapsack problem, which is defined as: given a set of items, each item has a mass and a value. We select some items and put them into a knapsack *K*, which maximizes the total value of *K*, and the total weight of *K* is less than or equal to the capacity of *K*. This can be interpreted as:

(10) $$\begin{array}{l} \text{max}\left(\text{value}\left(K\right)\right)\\ \begin{array}{cc} \text{subject to} & \text{mass}\left(K\right)\leq t_{c} \end{array} \end{array}$$

where *value*(*K*) and *mass*(*K*) denote the total value and total mass of selected items in *K*, and *t_c_* represents the capacity of *K*. Comparing optimization (9) and (10), we find that they are very similar to each other. The *value*(*K*) and *mass*(*K*) are much like the *acc*(*E*) and *D*(*E*) in our problem.

Nevertheless, our problem is not equivalent to the 0–1 knapsack problem. One of the main difference lies in the constraint interpretation, where the knapsack problem requires the total weight to be less than or equal to a given limit, and our problem requires the diversity of ensemble to be higher than or equal to *T_d_*. So that our problem more naturally converts to the 0–1 knapsack problem, we rewrite the constraint as: the inverse of the total diversity of *E* is less than or equal to the inverse of the diversity threshold *t_d_*.

Another difference is that the traditional 0–1 knapsack problem assumes that different items have different masses and weights, and hence the optimal solution is unique. However, in our case, since different base classifiers may have the same accuracy and diversity, multiple subsets of base classifiers may achieve the optimal solution simultaneously. Under such circumstances, to reduce the computational complexity of an ensemble, we select the subset with the least number of base classifiers as the final optimal solution.

Finally, our problem can be formulated as:

(11) $$\begin{array}{c} \begin{array}{l} \text{max}\left(\text{acc}\left(E\right)\right)\\ \begin{array}{cc} \text{subject to} & \frac{1}{D\left(E\right)}\leq \frac{1}{t_{d}} \end{array} \end{array}\\ \text{min}\left(\text{num}\left(E_{1}\right),\text{num}\left(E_{2}\right)\right),\text{where acc}\left(E_{1}\right)=\text{acc}\left(E_{2}\right) \end{array}$$

where *num*(*E*) denotes the number of base classifiers involved in *E*. The diversity threshold *t_d_* is defined by such a diversity, which the accuracy of an ensemble achieves at its highest value. As shown in Figure 9, such *t_d_* varies on different databases. Actually, the definition of *t_d_* depends on both the application requirement and data property. For applications with a high requirement on diversity, we should assign a relatively high value to *t_d_* (e.g., 0.9), and *vice versa*. In this paper, to make *t_d_* adapt to different databases, we define *t_d_* as the mean value of such *t_d_s* on four databases.

### 4.3. Base Classifier Integration

To aggregate the selected base classifiers *B*′ = {*b*_1_, *b*_2_, …, *b_L_*_′_}, we use the majority voting scheme. The ensemble classifies test samples by taking a majority vote among all base classifiers and assigns the class label that receives the largest vote to them.

### 4.4. Algorithm Description and Analysis

The skeleton of the proposed GL-E framework is illustrated in Algorithm 1.



**Algorithm: 1** Generic learning-based ensemble framework (GL-E).
**Input:** Gallery set 
$X^{G}=\left\{x_{ij}^{G};i=1,2,\ldots ,N,j=1,2,\ldots ,M_{G}\right\}$, probe set 
$X^{\mathbb{P}}=\left\{x_{i}^{\mathbb{P}};i=1,2,\ldots ,N*M_{P}\right\}$, parameter *L.***Output:** Decision vector of 
$X^{\mathbb{P}}:Y^{\mathbb{P}}=\left\{y_{i}^{\mathbb{P}};i=1,2,\ldots ,N*M_{P}\right\}$. **Initialization:**  Set 
$y_{i}^{\mathbb{P}}=0$; *i* = 1, 2, …, *N* * *M_P_*. **Step 1: Generic learning-based base classifier definition**  Generate a template image *f_T_* using Equation (1).  For *r* = 1, 2, …, *L*, repeat   **1.1**: Add a random matrix *R_r_* to *f_T_* to get a new image 
$f_{T\prime }^{r}$ defined by Equation (3);   **1.2:** Perform quad-tree decomposition on 
$f_{T\prime }^{r}$ to get an encoding pattern *P_r_* by Equation (4) and use *P_r_* to generate a new gallery dataset 
$X_{r}^{G}$;   **1.3:** Perform PCA + LDA on 
$X_{r}^{G}$ to define a base classifier *b_i_*, the output decision vector of *b_r_* is *Y_r_*, the accuracy of *b_r_* is denoted by *acc*(*b_r_*).  end **Step 2: Optimal selection:**  Select a subset of base classifiers *E* using the optimal solution in Equation (11). **Step 3: Majority voting:**  Integrate the base classifiers in *E* using the majority voting. **Output decision vector:**  Output the labels of samples in the probe set *Y* = *max*(Σ*Y_r_*).


We briefly analyze the computational complexity of GL-E according to its three main processing stages: generic learning-based base classifier definition, optimal base classifier selection and majority voting. The first stage mainly involves a template image calculation, quad-tree decomposition and the performance analysis of base classifiers based on PCA + LDA. Suppose a training set contains *m* samples, and the size of each face image is *d*. Both the generation of *f_T_*, $f_{T\prime }^{i}$ and quad-tree decomposition can be performed in linear time *O*(*d*). The classical PCA requires *O*(*d*^3^ + *d*^2^*m*) computations and LDA needs *O*(*mnt* + *t*^3^) [32], where *n* is the number of features and *t* = *min*(*m*, *n*). Since the feature dimension is usually smaller than that of the original face image, we have *d* > *n*. For an ensemble involving *L* base classifiers, the complexity of the first stage is summarized as *O*(*L*(*d*^3^ + *d*^2^*m*)). In the second stage, the tailored optimal 0–1 knapsack problem can be solved by dynamic programming, which requires *O*(*LlogL*) computations. The majority voting takes *O*(*L*) computations. Thus, the total computational complexity of our system is *O*(*L*(*d*^3^ + *d*^2^*m* + *LogL* + 1)).

## 5. System Performance Analysis

**Figure 10.**
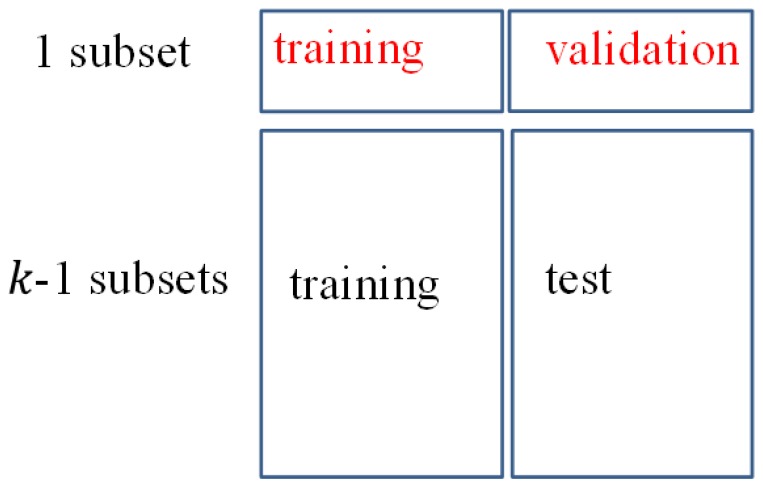
*k*-fold cross-validation for system performance evaluation.

We evaluated our system (GL-E) on four widely-used databases, namely: ORL [33], Extended Yale (Yale2) [34], AR [35] and FERET [36]. First, we analyzed the influences of a key parameter of our method: the number of base classifiers. Then, we compared our method with a wide range of face recognition algorithms on SSS and 15 state-of-the-art methods on the SSPP problem. Finally, we evaluated the performance of optimal base classifier selection, followed by the summary of simulation. To better compare our method with existing methods, we conduct the SSS face recognition with different numbers of enrolled samples as the training data. We randomly select *p* samples per subject from the database as training data and the rest of the samples as test data. We perform 10 splits for each database. For each split, we apply *k*-fold cross-validation (*k* = 5) to evaluate the performance of our algorithm with and without base classifier selection, respectively. Specifically, both the training data and test data are divided into *k* subsets, as shown in Figure 10. Each subset has the same number of subjects. The holdout validation is repeated *k* times. Each time, one of the *k* subsets is treated as training data and validation data, which is used to train the base classifier selection. The other *k* – 1 subsets are treated as training data and test data to report the performance of our method with and without base classifier selection (denoted by Our-Selection (‘Our-Sel.’) and Our-Original (‘Our-Org.’), respectively). The final performance of our method is calculated by the average accuracy over 10 * *k* trials (10 random splits by *k*-fold cross-validation). We use PCA + LDA to extract features and apply the nearest neighbor classification with the *L*_2_-norm for matching. The feature dimension of the PCA + LDA subspace is defined by *subject Number* – 1 for each database.

### 5.1. Databases

**Figure 11.**
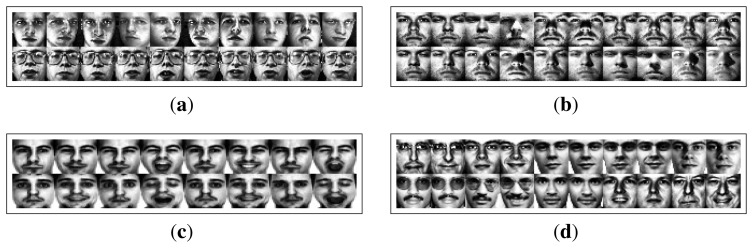
Four databases used in the experiments: (**a**) ORL; (**b**) Extended Yale (Yale2); (**c**) AR; (**d**) FERET.

- ORL database: This contains images from 40 subjects, with 10 different images per subject. Images vary on head poses, facial expressions, open or closed eyes, smiling or non-smiling, with glasses or no glasses and scale changes (up to about 10 percent).
- Extended Yale (Yale2) database: There are more than 20,000 single light source images of 38 subjects with 576 viewing conditions (nine poses in 64 illumination conditions). To evaluate the robustness of our method to the illumination changes, we use the 64 near frontal images under different illuminations per individual in the experiments.
- AR database: This contains over 4000 color face images of 126 people (70 men and 56 women), including frontal views of faces with different facial expressions, lighting conditions and occlusions (sunglasses and scarves). Each subject has 26 samples, taken in two sessions (separated by two weeks), and each session contains 13 samples. Similar to [11], eight subsets (A to H) of 800 images (eight images per subject) from 100 different subjects (50 men and 50 women) were used in our experiments. They were taken from two separate sessions and with different expressions. Figure 11c shows sample images from Subsets A to H, where A is used for training and the remaining seven subsets for testing.
- FERET database: This contains 13,539 facial images corresponding to 1565 subjects. Images differ in facial expression, ethnicity, gender and age. We worked with the grayscale images from GrayFERET (FERET Database, 2001). Two subsets (FERET-1 and FERET-2) are used to evaluate the performance of our method on SSS and SSPP face recognition, respectively. Similar to [7], FERET-1 contains all available subjects that have more than four frontal images. There are 665 images from 82 subjects in total. Similar to [11], FERET-2 is a subset containing 400 frontal face images belonging to 200 different people (71 females and 129 males). Each subject has two images (Fa and Fb) varying in race, gender, age, expression, illumination, scale, *etc*.

Face images in the first three databases are aligned to 32 × 32 using the method in [32]. The FERET database is normalized using the CSUFace Identification Evaluation System 5.1 [37]. The face images are cropped to the same size: 32 × 32. Sample images of all databases after histogram equalization are shown in Figure 11.

### 5.2. Parameter Analysis

**Figure 12.**
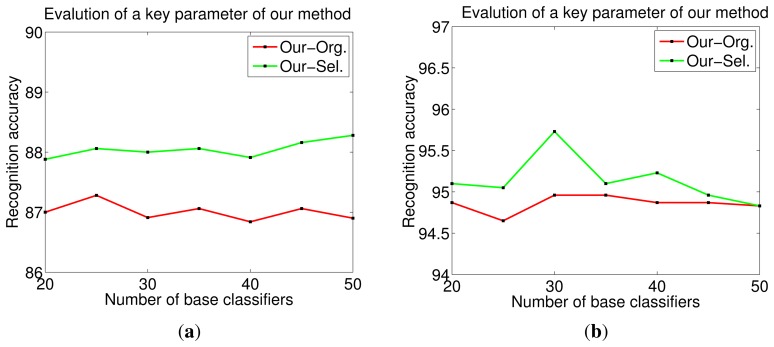
Influence of a key parameter (base classifier number) of our method on two databases: (**a**) ORL; (**b**) Yale2.

We investigate a key parameter of our method: the number of base classifiers *L*. We pick up two representative databases for analysis: a relatively large database, Yale2 with *p* = 5, and a small database, ORL with *p* = 2. The recognition accuracy of our method for these two databases, where *L* ranges from 20 to 50, is demonstrated in Figure 12a,b, respectively. We observe that GL-E has a stable recognition accuracy *versus* a large range of base classifiers for both databases. In order to save computational cost, *L* is set to a relatively small value, 20, without losing performance.

### 5.3. Comparison with Existing Methods on SSS Face Recognition

**Figure 13.**
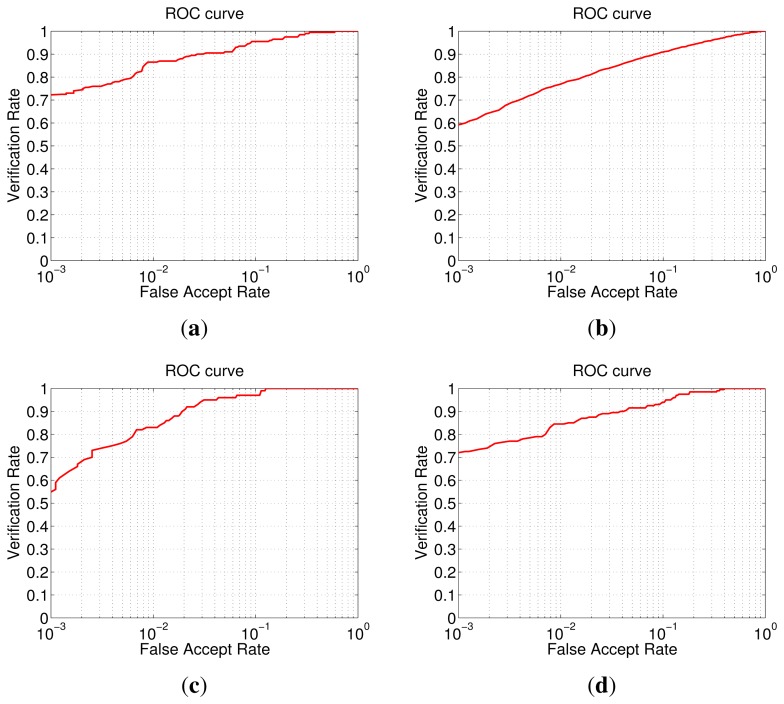
ROC curves of our method on four databases: (**a**) ORL (*p* = 2); (**b**) Yale2 (*p* = 5); (**c**) AR (subset B); (**d**) FERET-1.

In this part, we compared our method with a wide range of algorithms on SSS face recognition, including: conventional algorithms without ensemble and existing ensemble methods [5–11]. Tables 1, 2 and 3 tabulate the rank-one recognition accuracy of comparison methods on ORL, Yale2 and FERET-1 databases. In Tables 1, 2 and 3, the fist column lists the comparison methods, and the rest of the columns report the recognition accuracy of these methods using *p* samples per person as the training data. ROC curves of our method on four databases are shown in Figure 13.

#### 5.3.1. Comparison with Conventional Face Recognition Algorithms without Ensemble

We have compared our method with several conventional face recognition algorithms without ensemble, including: PCA family [7], LDA family [32], LPP family (locality preserving projections) [32], CCA family (canonical correlation analysis) [6] and several other representative methods, such as SVM, neural network-based methods (MLP (multilayer perception) and RBF (radial basis function network)) [7]. For a fair comparison, we reported the performances of these methods presented in their published papers. As these methods were not implemented on all of the databases in the reference papers, we compare with different methods on variant databases.

We got the following observations from Tables 1, 2 and 3:

(1) Performance degrades for the SSS problem: For all databases, we find that conventional methods obtain relatively high performance when the gallery data have moderate or a large number of training samples. However, their performances degrade as *p* decreases. This implies that the SSS problem challenges existing face recognition algorithms.
(2) Supervised learning outperforms unsupervised learning: Compared with PCA-based unsupervised learning methods, LDA-based supervised learning methods have relatively higher performance on almost all experiments, which shows the superiority of employing supervised learning in dealing with the SSS problem.
(3) Global and local feature-based methods have both advantages and disadvantages: For the ORL database, which contains multiple variations caused by head poses, facial expressions, open/closed eyes, head scale changes, with/without glasses, *etc.*, the local feature-based method (the classical LPP) outperforms the global feature-based method (the classical LDA). However, this is opposite for the Yale2 database, which contains illumination variations. This implies that both global- and local feature-based methods have their own advantages and disadvantages. Feature representation is not the key point in solving the SSS problem.
(4) Increasing diverse samples is more important: An interesting observation is captured between 2DCCA(in Tables 1 and 2) and 2DPCA (in Table 3). 2DCCA is an upgraded CCA algorithm targeting the SSS problem by directly extracting features from the image matrix rather than from the matrix to vector transformations. It consistently outperforms other variants of CCA in Tables 1 and 2. Similarly, 2DPCA applies this strategy, but it fails at performance improvement (see Table 3). This implies the 2D-matrix-based feature representation is not always effective for the SSS problem. The key point is to expand the face space by generating more samples rather than changing its representation.
(5) Our method performs better: Compared with all conventional methods without ensemble, our method, GL-E, obtains better performance on all experiments. There are two main reasons: (1) by generating new samples from GL-E, the original training data have been enlarged effectively, such that the face space can be represented by our method more accurately; (2) the generated base classifiers learned from the enlarged training data are diverse, offering complementary information for classification, and hence, the overall discriminative power is much greater than a single classifier. Benefiting from both new sample generation and ensemble learning, our method shows a better ability to address the SSS problem over conventional face recognition methods.

#### 5.3.2. Comparison with Existing Ensemble Methods

Ensemble methods have been widely applied to deal with SSS face recognition. We have compared our method with three kinds of representative methods: (1) global feature selection based on a random subspace [5]; (2) patch (block)-based local feature extraction, such as PCRC [9], and a patch-based method using LBP (local binary pattern) [7], which partition face images into small patches of the same size; and (3) global and local feature integration-based methods, such as the 30 region method [8], which defines 30 regions with large overlaps according to experimental experience, and the multi-scale patch-based method, MPCRC [9], which integrates the collaboration of patches of multi-scales to generate global feature representation. We reported the performance of LBP-based methods in [7]. We implemented the 30 region method and used the source code of PCRC and MPCRC in [9]. As this source code [9] requires all subjects to have the same number of training samples and test samples, only ORL and Yale2 met this requirement.

We got the following observations from Tables 1, 2 and 3:

(1) Ensemble learning can improve the performance: In Table 2, we observe that the ensemble-based methods outperform conventional algorithms without ensemble. This demonstrates the effectiveness of ensemble. However, existing ensemble methods are not always effective at coping with the SSS problem. For example, in Table 1, higher performances are achieved by the CCA and LPP families rather than existing ensemble algorithms. This shows the limited ability of existing methods when dealing with SSS.
(2) Local feature-based ensemble outperforms global feature-based ensemble: Compared with RS, the local patch-based method PCRC outperforms all conventional face recognition methods without ensemble of the Yale2 database (see Table 2) and has comparable results with S-LDA and S-LPP for the ORL database (see Table 1), which obtains the highest accuracy among conventional methods without ensemble. This shows the superiority of local feature extraction over global feature selection when dealing with local variations. However, the performance of patch-based methods is sensitive to the design of the patch size. An inappropriate patch size may lead to performance degradation, which has been illustrated in Table 3. Among the three variants of LBP, the one with a patch size of 7×7*w* performs better than the other methods at *p* = 2. However, this is not true for the other two versions (LPP 5 × 5 and LPP 7 × 7).
(3) Global and local feature integration improves performance further: To overcome the problem of patch-based methods, the global and local feature integration-based methods were proposed. We find that the 30 region method outperforms RS and PCRC in Table 2, and MPCRC obtains the highest performance among all existing methods in Tables 1 and 2. This implies the effectiveness of integrating local patches of variant sizes together to generate the global feature representation. However, we observe that the 30 region method performs worse than some of conventional methods for the ORL and FERET databases. The main reason is that the 30 regions are manually designed according to experimental experience after face registration. For well-registered databases, such as Yale2, which contains only near frontal images, the 30 region method performs well. However, for not well-registered databases, such as ORL and FERET, which contain variations of head poses, ethnicity, gender and age, its performance reduces obviously. We also find that MPCRC performs not so satisfactorily for the ORL database (see Table 1), which contains more variations. There are two main reasons: (1) it is developed on the small gallery data, while the probing set contains much more variations. Although the patch-based representation can deal with some local appearance variations, it is unable to reconstruct the expected face space with large variations caused by head poses *etc*.; (2) the multi-scale scheme of MPCRC just integrates the complementary information obtained at different scales of patches without considering the geometric co-relationship between local patches. According to the finding in [11], there is high overlap between manifolds of local patches. This suggests that the geometric information between local patches is also important for recognition.
(4) Our method performs better than existing ensemble methods: Compared with existing ensemble methods, our method obtains better performance in almost all experiments. It offers advantages over existing methods thanks to three main benefits: (1) Unlike existing methods, which train base classifiers from small training data, our method generates new samples to enlarge the given data. Base classifiers generated from the enlarged training data are more diverse and accurate for estimating the face space; (2) Other than existing methods, which divide face regions into separated patches, GL-E partitions face images into blocks of variant sizes according to a tree structure, which preserves the geometric correlationship between local blocks; (3) In contrast to existing methods, which involve all generated base classifiers for ensemble, our method formulates the ensemble as an optimal base classifier selection, which just selects appropriate base classifiers for integration. Our algorithm achieves a trade-off between diversity and accuracy. From Tables 1, 2 and 3, we can observe that Our-Sel. outperforms Our-Org. for almost all splits of databases, which demonstrates the effectiveness of base classifier selection.

### 5.4. Comparison with State-of-the-Art Methods for SSPP Face Recognition

We have compared our method with 15 state-of-the-art methods for SSPP face recognition, including PCA, (PC)^2^A [23], Enhanced (PC)^2^A (E(PC)^2^A) [38], 2DPCA [22], (2D)2PCA [24], Self-Organizing Map (SOM) [39], LPP [40], SVD-LDA [25], block PCA [27], block LDA [28], Uniform Pursuit (UP) [41], 30 region [8], MPCRC [9], ADA [10] and DMMA [12]. The methods, except 30 region [8], MPCRC [9] and ADA [10], were implemented by the authors of DMMA [12]. ADA utilized two additionally databases as a generic set: (1) Extended Multi Modal Verification for Teleservices and Security applications (XM2VTS); and (2) CAS-PEAL, which is developed in the Joint Research & Development Lab of Chinese Academy of Sciences (CAS) and the images of which are with different sources of variations, especially Pose, Expression, Accessories, and Lighting (PEAL). Since XM2VTS is not a public database, we cannot do experiments on it. Thus, we just reported its performance on the FERET-2 database presented in [10] for comparison. Table 4 tabulates the rank-one recognition rate of these methods for the AR and FERET-2 databases. We made the following five observations.

(1) Block-wise supervised learning does not always outperform the block-wise unsupervised learning: For the AR database, we find that the block-LDA does not perform better than block-PCA. This is because block-wise approaches assume that features are distributed in blocks evenly. Block-LDA partitions face images into multiple blocks and treats each block as an independent sample to estimate the within-class scatter matrix. However, the literature work [11] finds that there is high overlap between manifolds of local blocks. Summarizing separated blocks without considering any associations between them is not reliable to estimate the within-class matrix of LDA.
(2) The key point of virtual sample generation is to generate diverse samples: The virtual sample generation-based method, SVD-LDA, obtains the worst performance for the AR database. The reason is that by discarding just some smaller singular values of the original image, the virtually generated new samples are highly related to the original sample. Hence, the within-class scatter matrix cannot be accurately estimated under such cases. We draw the conclusion that generating new diverse samples, which have less co-relationship with the original samples, is the key point of applying virtual sample generation to take advantage of supervised learning.
(3) Generic learning needs further investigation: Compared with the generic learning-based method, ADA, we can see that both DMMA and GL-E achieve better performance than ADA. This further proves that generic learning needs to generate news samples other than grabbing samples from the generic set directly.
(4) Global and local feature-based ensemble reduces performances under SSPP: The performance of MPCRC and the 30 region method is not stable under SSPP. MPCRC degenerates to the original patch-based method, PCRC, without any collaboration of multiple patches of variant scales. As mentioned before, the performance of local patch-based methods is very sensitive to the design of patch size, especially when the database contains many local deformations. That is why it performs well on Subset E of the AR database, which contains just nearfrontal faces without any expressions, but degrades dramatically to 79% for the FERET-2 database, which contains a large number of local deformations caused by facial expressions *etc*. As explained before, the 30 region method reduces the performance for not well-registered databases. Thus, it performs well for part of AR database, which contains only frontal images, but not for the FERET-2 database.
(5) Our method shows advantages for SSPP: Among all of the comparison algorithms, GL-E outperforms most of the methods, and it obtains comparable performances with the recently developed DMMA algorithm for the AR database and outperforms DMMA with a gain in accuracy of 1.0 percent for the FERET-2 database. The reason why our algorithm is comparable to these state-of-the-art methods is that GL-E does expand the face space in the training stage by generating new samples. Compared with existing virtual sample generation methods, the new generated samples are quite diverse and different from the original training data, thanks to the introduction of random matrices. Moreover, our method not only encodes discriminant features, but also geometric information, which contribute to recognition. We acknowledge that DMMA outperforms our method on some subsets of the AR database. This is mainly because it extracts features in a person-specific manner rather than in a generic way. By modeling the manifold surface for each subject and performing classification through maximizing the manifold margin between different subjects, it employs personal characteristics (e.g., age, hair style, *etc*.) during recognition and improves the recognition accuracy. However, we argue that the effectiveness of this strategy is challenged by the number of subjects involved. With the increase of the number of subjects in the database, the maximization of the manifold margins between different subjects becomes more difficult. This then causes errors. That is why DMMA has a reduced performance for the FERET-2 database, which contains twice the subjects of the AR database.

### 5.5. Evaluation of the Optimal Base Classifier Selection

**Figure 14.**
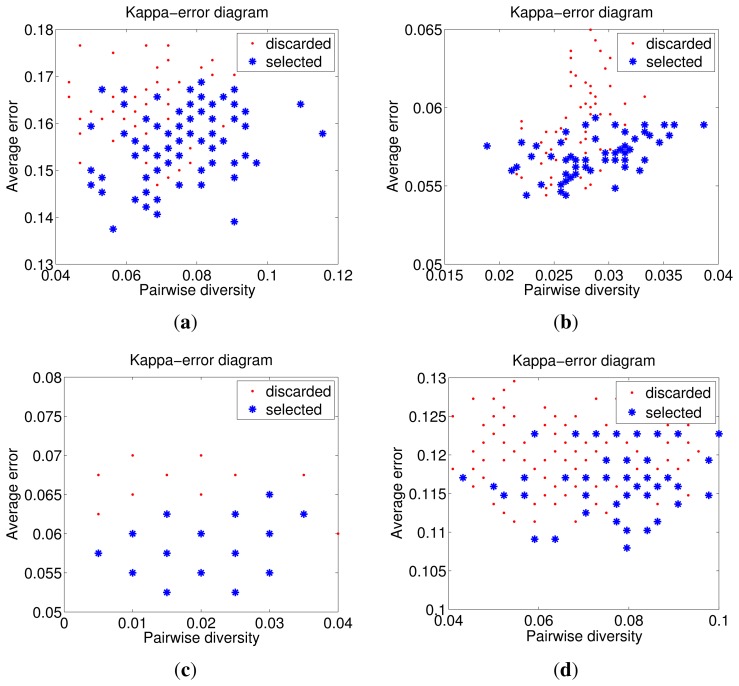
Kappa-error diagrams of four databases (red points denote the discarded base classifiers, blue stars represent the selected base classifiers): (**a**) ORL (*p* = 2); (**b**) Yale2 (*p* = 5); (**c**) AR (Subset B); (**d**) FERET-1 (*p* = 2).

In order to better demonstrate the effectiveness of base classifier selection, the kappa-error diagram [42] is used for illustration, which visualizes the pairwise diversity of base classifiers against their averaged error. In Figure 14, the *x*-axis is the diversity *div* between pairs of base classifiers and the *y*-axis is the averaged individual error *Err* of pairwise classifiers. The less the *Err* is, the higher the accuracy becomes. The most desirable pairs of classifiers lie in the bottom right side, where the diversity *div* is high and the *Err* is low. For a better demonstration, we use different colors and marks to differentiate base classifiers. The red points represent discarded base classifiers, and the blue stars denote selected base classifiers. From the kappa-error diagrams in Figure 14, we can see that the number of base classifiers and the average errors are reduced obviously after selection. As expected, selected base classifiers are mainly located at the right bottom side in the figure. This verifies the effectiveness of the optimal selection in alleviating the diversity/accuracy dilemma.

### 5.6. Summary of Simulation

In this section, we have studied the influence of a key parameter, evaluated the performance of our system on both SSS and SSPP face recognition by comparing with a wide range of existing algorithms and analyzed the effectiveness of base classifier selection. From the experimental results, we find that our method performs well on a large range of base classifiers. Compared with conventional algorithms on SSS face recognition, we observe that ensemble learning improves the recognition accuracy, thanks to the base classifier collaboration. Benefiting from both new sample generation and ensemble learning, our method outperforms existing ensemble methods, whether with or without ensemble. Moreover, our base classifiers encode not only discriminant features, but also the geometric correlationship between subregions. For SSPP face recognition, our method performs better than almost all 15 state-of-the-art methods, and it obtains comparable performances with the latest developed DMMA algorithm, which demonstrates the superiority of our system in dealing with both SSS and SSPP problems. Through evaluation of the optimal base classifier selection using kappa-error diagrams, we find that the number of base classifiers and the average errors are reduced obviously after selection. Our system effectively alleviates the diversity/accuracy dilemma.

## 6. Conclusions

In multi-camera networks, person re-identification is an essential and challenging task. It often uses faces as a distinct trial and suffers from the small sample size (SSS) problem arising from the small number of training samples compared to the high dimensionality of the sample space. In this paper, we propose a novel generic learning-based ensemble framework (GL-E) to address this problem. GL-E overcomes the two serious problems of existing ensemble methods for SSS face recognition: (1) base classifiers are not diverse enough using small training data; and (2) the diversity/accuracy dilemma occurs during ensemble. We solve the first problem by generating more diverse base classifiers from the enlarged training data using generic learning. Additionally, the second problem is settled by applying an optimal base classifier selection, which selects a subset of appropriate base classifiers for aggregation. Use of this solution achieves the trade-off between the diversity and the accuracy of an ensemble. Extensive experimental results on four widely-used databases demonstrate that GL-E estimates a more accurate and robust ensemble for both SSS and SSPP face recognition.

Though promising results have been achieved, the proposed system still has space to extend. For instance, we adopt random matrices in generating base classifiers; random matrices may introduce some noises, which influence the quad-tree decomposition. How to remove such noises needs further investigation. Moreover, diversity is an important factor to evaluate an ensemble system. We simply use a common definition, the “disagreement degree”, in this paper. There should be better schemes to investigate this factor. For the future work of this paper, we are going to improve the system with respect to these issues.

## Figures and Tables

**Table 1.** Evaluation of the ORL database. MPCRC, multi-scale patch-based collaborative representation. LLP, locality preserving projections; CCA, canonical correlation analysis.

**Method**		***p*** = 2	***p*** = 3	***p*** = 4	***p*** = 5
PCA		66.9	76.6	82.1	86.3

LDA family	LDA	72.5	84.0	89.4	92.8
	PCA + LDA	77.7	86.1	90	92.7
	R-LDA	79.1	89.0	93.7	96.4
	S-LDA	82.9	91.9	95.9	97.7

CCA family	PCA + CCA	81.3	87.5	89.2	91.8
	CCA + Perturbation	81.3	87.8	89.5	92.7
	KPCA+ CCA	81.5	88.8	92.8	93.5
	2DCCA	85.0	89.5	93.3	95.0

LPP family	LPP	78.0	86.2	90.3	93.2
	R-LPP	79.1	89.1	93.6	96.4
	S-LPP	82.9	91.9	95.9	97.7
	OLPP	79.5	89.2	93.6	96.2

Ensemble	RS	76.8	83.6	87.8	93.3
	PCRC	70.6	81.8	87.9	89.5
	MPCRC	78.4	84.3	87.1	91.5
	30Region	80.6	87.8	90.7	94.8

	Org.	86.3	92.2	95.4	97.5
Our	Sel.	**87.1**	**93.1**	**96.2**	**98.1**

**Table 2.** Evaluation of the Yale2 database.

**Method**		***p*** = 5	***p*** = 10
PCA		36.4	53.6

LDA family	LDA	75.5	87.5
	PCA + LDA	76.3	87.0
	R-LDA	77.2	89.6

CCA family	PCA + CCA	73.0	86.0
	CCA + Perturbation	73.0	87.0
	KPCA + CCA	75.0	88.0
	2DCCA	87.5	91.5

LPP family	LPP	67.9	81.5
	Tensor-LPP	71.7	82.9
	OLPP	71.6	83.7

Ensemble	RS	84.3	95.8
	PCRC	91.0	98.8
	MPCRC	92.8	**99.1**
	30Region	90.3	97.8

	Org.	95.0	98.8
Our	Sel.	**95.5**	98.9

**Table 3.** Evaluation of the FERET-1 database. RPF, radial basis function; LBP, local binary pattern.

**Method**		***p*** = 2	***p*** = 3	***p*** = 4
PCA family	PCA	82.4	86.6	89.4
	2DPCA	81.9	86.4	89.2
	KPCA	82.3	87.8	91.6

SVM family	SVM	68.8	91.7	95.1
	PCA + SVM	91.5	95.8	97.2

Neural Networks	MLP	72.9	83.4	85.9
	RBF	85.3	93.2	96.8

Ensemble	RS	75.7	81.5	85.7
	LBP-5 × 5	89.4	92.1	94.2
	LBP-7 × 7	91.5	94.4	96.0
	LBP-7 × 7w	**92.9**	95.1	96.6
	30 Region	73.0	92.3	82.1

	Org.	85.1	96.9	96.4
Our	Sel.	91.9	**96.9**	**98.2**

**Table 4.** Evaluation of the AR and FERET-2 databases for single sample per person (SSPP) face recognition. DMMA, discriminative multi-manifold analysis; ADA, adaptive discriminant analysis.

**Method**	**AR**							**FERET**	**Year**

	**B**	**C**	**D**	**E**	**F**	**G**	**H**		
PCA	97	87	60	77	76	67	38	84.0	1991
(PC)^2^A	97	87	62	77	74	67	40	84.5	2002
E(PC)^2^A	97	87	63	77	75	68	41	85.5	2004
2DPCA	97	87	60	76	76	67	37	84.5	2004
(2D)^2^PCA	98	89	60	71	76	66	41	85.0	2005

SOM	98	88	64	73	77	70	42	91.0	2005
LPP	94	87	36	86	74	78	20	84.0	2005
SVD-LDA	73	75	29	75	56	58	19	85.5	2005
Block PCA	97	87	60	77	76	67	38	84.5	2004
Block LDA	85	79	29	73	59	59	18	86.5	2004
UP	98	88	59	77	74	66	41	90.0	2010
30 region	91	94	37	91	66	81	22	86.0	2012
MPCRC	87	95	25	**96**	80	**88**	9	79.0	2012

ADA	N/A	N/A	N/A	N/A	N/A	N/A	N/A	92.6	2013
DMMA	**99**	93	**69**	88	**85**	79	45	93.0	2013

Our-Sel.	98	**96**	55	90	83	80	**48**	**94.0**	2014
